# Physical Function and Daily Living in Postmenopausal Women with Osteoporotic Vertebral Fractures: A Cross-Sectional Study

**DOI:** 10.3390/biomedicines14081765

**Published:** 2026-08-05

**Authors:** Ngoc Quyen Nguyen, Hong Van Vu, Viet Ha Pham, Thi Thu Thuy Nguyen

**Affiliations:** 1 Department of General Outpatient Clinic, 108 Military Central Hospital, No. 1 Tran Hung Dao Street, Hai Ba Trung Ward, Hanoi 100000, Vietnam; vuhongvan38@gmail.com (H.V.V.); thuyamy108@gmail.com (T.T.T.N.); 2 Department of Surgery, Hai Phong International General Hospital, No. 124 Nguyen Duc Canh Street, Le Chan Ward, Hai Phong 180000, Vietnam; vietha6@yahoo.com

**Keywords:** osteoporotic vertebral fracture, postmenopausal osteoporosis, activities of daily living, Barthel Index, Lawton IADL, pain, functional disability

## Abstract

**Background/Objectives:** Osteoporotic vertebral fractures (OVFs) can cause persistent pain and loss of independence. This study quantified impairment in basic and instrumental activities of daily living and identified independent cross-sectional correlates in postmenopausal women with OVFs. **Methods:** This cross-sectional analysis included 184 postmenopausal women from a 200-patient OVF cohort at a spine clinic in Vietnam. Functional status was assessed using the Barthel Index (BI) and Lawton Instrumental Activities of Daily Living (IADL) scale. Primary analyses used multivariable linear regression with HC3 robust inference; ordered-logistic and model-specification analyses assessed robustness. **Results:** Mean age was 69.95 ± 7.78 years and mean visual analog scale (VAS) pain score was 6.69 ± 1.58. Basic-activity dependence was present in 81.5% of participants (mean BI, 82.96 ± 16.45), and 66.8% had at least one IADL limitation (mean IADL, 5.84 ± 2.04). In the adjusted BI model, higher VAS pain intensity was the strongest observed correlate of poorer function (B = −7.996; β = −0.769; *p* < 0.001), and higher body mass index was associated with lower BI. In the adjusted IADL model, higher VAS pain intensity (B = −0.694; β = −0.538; *p* < 0.001) and older age were associated with lower scores. Ordinal sensitivity analyses confirmed the pain association, whereas the BMI finding was attenuated. **Conclusions:** Pain intensity was the strongest observed cross-sectional correlate of both functional domains. The findings support comprehensive functional assessment and hypothesis-driven longitudinal studies, but they do not establish that pain reduction will cause functional recovery.

## 1. Introduction

Osteoporotic vertebral fractures (OVFs) are the most prevalent fracture complication of postmenopausal osteoporosis and may occur without major trauma or remain clinically unrecognized. Nevertheless, their consequences can be cumulative, including persistent back pain, progressive vertebral collapse and kyphosis, impaired balance, reduced mobility, and increasing dependence in daily life [1,2,3].

The pathway from vertebral fracture to disability is not determined by bone loss alone. Vertebral collapse and kyphotic posture alter sagittal alignment and increase the demand on spinal extensor muscles, while pain-related guarding and avoidance of movement may accelerate deconditioning. Age-related loss of muscle strength and physical performance can further impair balance, mobility, and independence. These interacting skeletal, postural, and muscular factors provide a plausible bone–muscle framework linking OVF to functional decline [4,5,6,7,8].

Functional impairment after OVF spans two complementary domains. Basic activities of daily living (ADL), measured by the Barthel Index (BI), capture fundamental self-care capacity, including feeding, bathing, dressing, toileting, transfers, and mobility [9]. Instrumental activities of daily living (IADL), measured by the Lawton IADL scale, capture more complex competencies required for independent community living, including meal preparation, shopping, transportation, and medication and financial management [10]. Concurrent assessment of both domains may provide a broader functional profile than either instrument alone. The global disability burden associated with osteoporotic fractures further supports evaluation across complementary functional domains [11].

Pain intensity, age, body mass index (BMI), fracture burden, muscle reserve, and socioeconomic factors may contribute to functional impairment, but their independent effects remain inconsistently characterized. Previous OVF research has commonly focused on pain, disability, and quality of life rather than simultaneously modeling basic and instrumental ADL outcomes [12].

This study aimed to: (1) describe the distribution of BI and IADL scores and (2) identify independent cross-sectional correlates of BI and IADL scores in postmenopausal women with OVFs. By distinguishing correlates of basic and instrumental disability, the study also sought to inform future multidisciplinary research integrating fracture care, pain assessment, and functional rehabilitation.

## 2. Materials and Methods

### 2.1. Study Design and Setting

This cross-sectional study was conducted at the Spine Outpatient Clinic, Department of Genaral Out-Patient Clinic, 108 Military Central Hospital, Hanoi, Vietnam, from 13 February 2025 to 8 December 2025, and is reported in accordance with the STROBE guidelines for cross-sectional studies [13].

### 2.2. Participants

**Figure 1:**
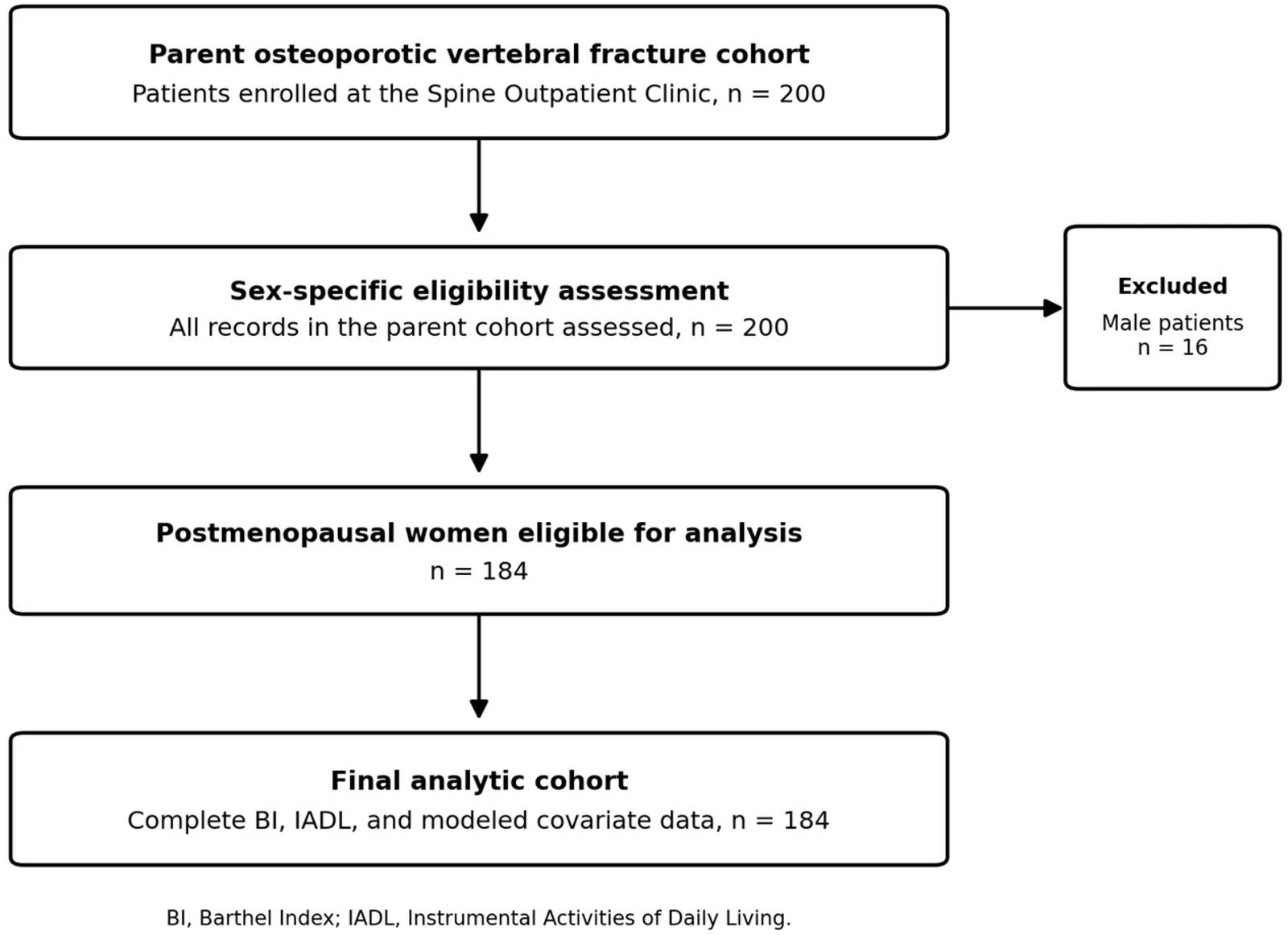
Flow diagram of participant inclusion in the sex-specific analytic cohort. The parent cohort comprised 200 patients with osteoporotic vertebral fractures. Sixteen male patients were excluded, leaving 184 postmenopausal women with complete BI, IADL, and modeled covariate data for the primary and robustness analyses.

Patients were enrolled by convenience sampling during routine clinic visits into a fixed parent cohort of 200 patients with OVFs. The parent cohort was an available clinical cohort rather than a population-based consecutive registry, and a screening denominator for potentially eligible clinic attendees who were not enrolled was not available. The present sex-specific analysis included all 184 postmenopausal women in the parent cohort; 16 male patients were excluded (Figure 1). Inclusion criteria were age ≥ 50 years; confirmed osteoporosis (DXA T-score ≤ −2.5 at the lumbar spine, femoral neck, or total hip); radiologically confirmed vertebral fracture; postmenopausal status (≥12 months of amenorrhea); ability to participate; and written informed consent. Exclusion criteria were fracture due to malignancy, high-energy trauma, or non-osteoporotic causes; severe cognitive impairment; current inpatient status; or recent spinal surgery.

### 2.3. Measurements

Sociodemographic data included age, BMI (kg/m^2^), education level, socioeconomic status (low/middle/high), and residential area. Age was calculated as 2025 minus the recorded year of birth. Medical history included hypertension, diabetes mellitus, rheumatoid arthritis, corticosteroid use, and self-reported use of analgesic products for which the exact name and dose were not documented. Menopause duration was recorded. Pain intensity was assessed using a 0–10 VAS. Bone mineral density was measured using a Discovery Wi dual-energy X-ray absorptiometry system (Hologic, Inc., Marlborough, MA, USA; software version 13.5.4), and T-scores were recorded at the femoral neck and lumbar spine (L1–L4). Radiologically confirmed fractures were recorded by vertebral level and summarized by location and number. Formal Genant semiquantitative severity grades [14], fracture acuity, and time since fracture were not systematically available and were not analyzed. The database did not contain standardized fields for anti-osteoporosis treatment, calcium or vitamin D use, previous fractures, neurological disease, sarcopenia, or rehabilitation exposure.

Basic ADL was assessed using the BI (0–100; higher = more independent). Dependence levels were categorized as fully independent (100), mild dependence (91–99), moderate dependence (61–90), severe dependence (21–60), and total dependence (0–20) [9]. Instrumental ADL was assessed using the Lawton IADL scale (0–8; higher = more independent) [10]. For descriptive presentation only, IADL scores were grouped as 8, 6–7, and 0–5; these groups were not interpreted as validated severity categories. Vietnamese-language interviewer-administered forms prepared from the original instruments were used by trained nursing researchers. Formal cultural validation of these exact Vietnamese forms in women with OVFs was not available; therefore, internal consistency was estimated post hoc using Cronbach’s alpha and the absence of formal language validation was treated as a limitation.

### 2.4. Statistical Analysis

Data were analyzed using Python 3.13.5 (NumPy 2.3.5, SciPy 1.17.0, and statsmodels 0.14.6). Continuous variables are presented as mean ± standard deviation and categorical variables as frequency and percentage. BMI and pain categories were derived from continuous BMI and VAS values. Education was dichotomized as primary education versus secondary education or higher because the two upper categories were sparse. Socioeconomic status and rheumatoid arthritis were described but not modeled because the extreme socioeconomic groups and the rheumatoid arthritis subgroup were small. Binary predictors were coded as no/yes; residential area as rural/urban; fracture location using lumbar fractures as the reference; and number of fractured vertebrae using one fracture as the reference. The 16 predictors were specified before model fitting on clinical grounds and data availability; univariable *p* values were not used for variable selection. All predictors were entered simultaneously into the primary multivariable linear models. Because BI and IADL are bounded and residual normality was imperfect, HC3 heteroskedasticity-robust standard errors, confidence intervals, and *p* values were used. There were 11.5 participants per predictor; a sensitivity calculation indicated that n = 184 provided 80% power at α = 0.05 to detect an overall effect size of f^2^ ≥ 0.113 for a 16-predictor model. Secondary analyses included proportional-odds models using four ordered BI categories (≤60, 61–90, 91–99, and 100) and the exact IADL score (0–8), models with and without VAS because pain may lie on the pathway between fracture and disability, quadratic terms for age, BMI, and VAS, joint tests of VAS-by-fracture-location/count interactions, and influence analyses using Cook’s distance. Continuous predictors were standardized in the ordered models. Statistical significance was set at *p* < 0.05 (two-tailed).

### 2.5. Ethical Considerations

The study was conducted in accordance with the Declaration of Helsinki and approved by the Institutional Review Board of Thang Long University, Hanoi, Vietnam (protocol code 25021302/QĐ-ĐHTL; approval date: 12 February 2025). Written informed consent was obtained from all participants. Data were anonymized, and no study-specific invasive procedures were performed.

## 3. Results

### 3.1. Sample Characteristics

Table 1 presents participant characteristics. Mean age was 69.95 ± 7.78 years and mean BMI was 21.54 ± 3.27 kg/m^2^; 15.8% were underweight. Most participants lived in rural areas (79.3%), had middle socioeconomic status (92.9%), and had completed secondary education (62.0%). Hypertension was the most common comorbidity (36.4%), and 58.2% reported use of undocumented analgesic products. Mean VAS pain intensity was 6.69 ± 1.58; 58.2% had severe pain (VAS ≥ 7). Lumbar fractures were most frequent (47.8%), followed by thoracolumbar (32.6%) and thoracic fractures (19.6%). One fractured vertebra was recorded in 46.7%, two in 21.2%, and three or more in 32.1%.

Mean BI was 82.96 ± 16.45; 18.5% were fully independent, 14.1% had mild dependence, 54.3% had moderate dependence, 12.5% had severe dependence, and 0.5% had total dependence. Overall, 81.5% had some degree of dependence. Mean IADL was 5.84 ± 2.04; 33.2% scored 8, 28.3% scored 6–7, and 38.6% scored 0–5. Internal consistency was good for both instruments (Cronbach’s α = 0.873 for BI and 0.812 for IADL).

### 3.2. Univariable Regression

Table 2 and Table 3 present the univariable analyses using HC3 robust inference. VAS pain intensity was the strongest correlate of both BI (B = −8.115; R^2^ = 0.608; *p* < 0.001) and IADL (B = −0.840; R^2^ = 0.424; *p* < 0.001). Older age, lower education, lower femoral-neck and lumbar-spine T-scores, thoracolumbar rather than lumbar fracture location, three or more rather than one fractured vertebra, and longer menopause duration were also associated with poorer BI and IADL in univariable models. These associations were reassessed in the multivariable models.

### 3.3. Multivariable Regression—Barthel Index

Table 4 presents the multivariable BI model (R^2^ = 0.661; adjusted R^2^ = 0.628; all VIFs ≤ 4.41). Higher BMI was associated with lower BI (B = −0.675; 95% CI −1.265 to −0.084; β = −0.134; *p* = 0.025). VAS pain intensity was the strongest observed cross-sectional correlate (B = −7.996; 95% CI −9.534 to −6.459; β = −0.769; *p* < 0.001), with the linear coefficient representing an average association across the observed pain range. The age association was attenuated after robust adjustment (*p* = 0.065), and the other included variables were not independently significant.

### 3.4. Multivariable Regression—Lawton IADL

Table 5 presents the multivariable IADL model (R^2^ = 0.531; adjusted R^2^ = 0.486; all VIFs ≤ 4.41). Older age was associated with lower IADL (B = −0.082; 95% CI −0.143 to −0.022; β = −0.314; *p* = 0.008). VAS pain intensity was the strongest observed cross-sectional correlate (B = −0.694; 95% CI −0.845 to −0.542; β = −0.538; *p* < 0.001). Education, BMI, residential area, comorbidities, medication-related variables, DXA T-scores, fracture location and burden, and menopause duration were not independently significant.

### 3.5. Sensitivity and Robustness Analyses

In ordered-logistic sensitivity analyses, VAS remained strongly associated with lower functional status for both outcomes. Older age remained associated with lower IADL, whereas the BMI–BI association was attenuated (*p* = 0.093). Because the ordered BI model produced some secondary associations that were not consistent with the linear model, these findings were interpreted as model-specification sensitivity rather than replacement primary estimates (Table 6). Models omitting VAS did not make fracture location or fracture number statistically significant for BI or IADL (all *p* > 0.22), indicating that the absence of fracture associations was not explained solely by inclusion of pain (Table 7).

Exploratory quadratic terms suggested nonlinearity for age and VAS in both outcomes, whereas evidence for BMI nonlinearity was absent for BI (*p* = 0.220) and borderline for IADL (*p* = 0.060). Joint tests of VAS-by-fracture-location/count interactions were not significant (BI *p* = 0.360; IADL *p* = 0.587). Twelve BI observations and ten IADL observations exceeded the conventional Cook’s distance threshold of 4/n, but the maximum values were 0.146 and 0.065, respectively, and none approached 1. Excluding these observations preserved the strong VAS associations and the age–IADL association; the BMI–BI association also remained statistically significant (Table 8).

## 4. Discussion

This study characterized functional impairment in 184 postmenopausal Vietnamese women with OVFs and identified domain-specific cross-sectional correlates. Pain intensity showed the strongest and most consistent association with both functional domains. Higher BMI was associated with poorer basic ADL in the primary linear model, although this association was attenuated in the ordinal sensitivity analysis; older age was consistently associated with poorer instrumental ADL.

The mean BI of 82.96 and the finding that 81.5% of participants had at least some dependence indicate that many women retained partial self-care capacity but experienced clinically relevant limitations. The mean IADL score of 5.84 and an impairment prevalence of 66.8% further show that more complex community-based tasks were frequently affected. This pattern is consistent with longitudinal evidence showing that pain, disability, and quality of life may remain impaired for months after an acute vertebral fragility fracture [12]. Assessing BI and IADL together is therefore clinically useful: BI identifies limitations in essential self-care and mobility, whereas IADL can reveal losses of autonomy that may not be evident from basic ADL alone.

Pain showed the strongest observed association with both BI and IADL. In the primary linear model, each one-unit increase in VAS corresponded to an average 8.0-point lower BI score; however, the quadratic analysis indicated that this slope was not constant across the entire pain range. Nociceptive input can arise from the fractured vertebral body and surrounding tissues, while muscle spasm and altered spinal loading may perpetuate symptoms. Pain can also restrict walking, transfers, exercise, and social participation, creating a cycle of inactivity and deconditioning [2,12]. Pain may therefore be part of the pathway between fracture and disability rather than a conventional confounder. Models that omitted VAS did not reveal independent fracture-location or fracture-number associations, but the cross-sectional design cannot establish mediation, directionality, or whether reducing pain would improve function.

The strong pain–function relationship also has a plausible bone–muscle basis. Vertebral collapse and kyphotic deformity shift the trunk anteriorly and increase the demand placed on spinal extensor muscles [4]. Pain-related guarding and reduced activity may then diminish strength, endurance, balance, and postural control. Age-related sarcopenia further reduces muscle strength and physical performance and is clinically linked to disability and falls [5]. Exercise guidance for people with osteoporosis or vertebral fracture therefore emphasizes individualized resistance, balance, posture, and functional training [6,7]. Although muscle mass and composition were not measured in our cohort, these established postural and muscular pathways provide a biologically coherent framework for the observed importance of pain and age.

Older age was associated with lower IADL, although exploratory analysis suggested that the age–function relationship may be nonlinear. Instrumental tasks require greater cognitive, sensory, balance, and executive reserve than basic self-care and may therefore be more sensitive to age-related multimorbidity, sarcopenia, and reduced physiological resilience. Higher BMI was associated with lower BI in the primary model but not with IADL, and its BI association was weaker in the ordinal sensitivity analysis. BMI does not distinguish fat mass from lean mass, only two participants had obesity, and the result should not be interpreted as evidence that weight reduction would improve function.

Education was associated with both functional outcomes in univariable analysis but was not independently significant after adjustment, suggesting that the crude association was partly explained by age, pain, and other clinical factors. Socioeconomic context can influence instrumental activities through access to transportation, financial resources, medication support, and family or community assistance [15]. However, 92.9% of this cohort was classified as middle socioeconomic status, while the low- and high-status groups contained only six and seven participants. Socioeconomic status was therefore not modeled because estimates based on these sparse groups would be unstable and potentially misleading. Social barriers remain clinically relevant, but this dataset cannot support a reliable estimate of their independent effect.

Neither BMD nor the available fracture-location and fracture-number variables were independently associated with function after adjustment, and these variables remained non-significant in models that omitted pain. This does not imply that skeletal fragility or fracture severity is unimportant. BMD contributes to fracture susceptibility, and vertebral deformity may alter spinal mechanics, but disability may also be shaped by pain, age-related physiological reserve, adaptation, and social support [1,4,5]. Formal Genant grade, fracture acuity, and time since fracture were unavailable; consequently, the analysis could not determine whether active, recent, or severe fractures had stronger functional effects.

Menopause duration was associated with both outcomes in univariable analysis but lost significance after adjustment. Its close relationship with chronological age suggests that duration since menopause may primarily reflect cumulative aging and skeletal exposure rather than exerting an independent effect on post-fracture function.

The findings have practical hypothesis-generating implications. In clinical practice, fracture and osteoporosis assessment may be complemented by repeated pain assessment and evaluation of BI and IADL. Consensus recommendations and systematic-review evidence support appropriately tailored resistance, balance, posture, and functional exercise for people with osteoporosis or osteoporotic vertebral fracture, while a randomized trial reported benefits from low-intensity back exercise [6,7,8]. Exercise should be staged according to fracture acuity, pain severity, balance, and neurological status.

Future longitudinal studies should record fracture date, imaging evidence of acuity, formal severity grade, and changes in pain and function over time to test whether pain mediates recovery. Detailed information on analgesic type and dose, anti-osteoporosis medication, calcium and vitamin D use, previous fractures, neurological disease, rehabilitation exposure, appendicular muscle mass, grip strength, gait speed, paraspinal muscle composition, sagittal alignment, and biological markers would allow more complete control of confounding and direct testing of the proposed bone–muscle pathway.

This study has several limitations. Its cross-sectional design precludes causal inference and permits reverse causality: greater disability could increase pain reporting and inactivity, while pain could also worsen function. Residual confounding is likely because fracture acuity, time since fracture, formal Genant severity, previous fractures, neurological disease, detailed medication and osteoporosis treatment, calcium or vitamin D use, sarcopenia, and rehabilitation exposure were not recorded. Convenience sampling at a single tertiary military hospital and the absence of a screening denominator limit generalizability and prevent estimation of participation bias. BI and IADL were administered face to face by trained nursing researchers, so interviewer and social-desirability bias cannot be excluded. The exact Vietnamese-language forms had not undergone formal cultural validation in women with OVFs, although internal consistency was good in this sample. The low- and high-socioeconomic-status groups and the rheumatoid arthritis subgroup were too small for stable estimates, and upper education categories were combined. IADL items may be influenced by gender roles and household arrangements. BI and IADL are bounded scales with ceiling effects; robust linear models were supplemented with ordered-logistic analyses, but no single model fully resolves these measurement limitations. Finally, exploratory nonlinear and influence analyses were post hoc and should be confirmed in an independent cohort.

## 5. Conclusions

Postmenopausal women with OVFs experienced substantial impairment in both basic and instrumental activities of daily living. Pain intensity was the strongest observed cross-sectional correlate of both domains. Higher BMI was associated with poorer BI in the primary linear model but was attenuated in ordinal sensitivity analysis, whereas older age was consistently associated with poorer IADL. These findings support comprehensive functional assessment and longitudinal research integrating fracture characteristics, pain, muscle status, treatment exposure, and rehabilitation. They do not establish that modifying pain, BMI, or age-related factors will cause functional recovery.

## Figures and Tables

**Table 1:** Participant characteristics and functional status.

Characteristic	n (%)	Mean ± SD
Age (years)		69.95 ± 7.78
50–59 years	15 (8.2%)	
60–69 years	83 (45.1%)	
70–79 years	67 (36.4%)	
≥80 years	19 (10.3%)	
BMI (kg/m^2^)		21.54 ± 3.27
Underweight (<18.5)	29 (15.8%)	
Normal (18.5–24.9)	133 (72.3%)	
Overweight (25.0–29.9)	20 (10.9%)	
Obesity (≥30.0)	2 (1.1%)	
Residential area		
Rural	146 (79.3%)	
Urban	38 (20.7%)	
Socioeconomic status		
Low	6 (3.3%)	
Middle	171 (92.9%)	
High	7 (3.8%)	
Education		
Primary	62 (33.7%)	
Secondary	114 (62.0%)	
Intermediate	5 (2.7%)	
College/university	3 (1.6%)	
Hypertension—yes	67 (36.4%)	
Rheumatoid arthritis—yes	6 (3.3%)	
Diabetes mellitus—yes	23 (12.5%)	
Corticosteroid use—yes	10 (5.4%)	
Undocumented analgesic product use—yes	107 (58.2%)	
Pain intensity (VAS, 0–10)		6.69 ± 1.58
No/mild pain (0–3)	6 (3.3%)	
Moderate pain (4–6)	71 (38.6%)	
Severe pain (≥7)	107 (58.2%)	
T-score, femoral neck		−2.82 ± 0.80
T-score, lumbar spine		−3.29 ± 0.77
Fracture location		
Thoracic	36 (19.6%)	
Lumbar	88 (47.8%)	
Thoracolumbar	60 (32.6%)	
Number of fractured vertebrae		
One	86 (46.7%)	
Two	39 (21.2%)	
Three or more	59 (32.1%)	
Menopause duration (years)		21.78 ± 9.05
Barthel Index (0–100)		82.96 ± 16.45
Fully independent (100)	34 (18.5%)	
Mild dependence (91–99)	26 (14.1%)	
Moderate dependence (61–90)	100 (54.3%)	
Severe dependence (21–60)	23 (12.5%)	
Total dependence (0–20)	1 (0.5%)	
Lawton IADL (0–8)		5.84 ± 2.04
Score 8 (full independence)	61 (33.2%)	
Score 6–7	52 (28.3%)	
Score 0–5	71 (38.6%)	

Data are presented as n (%) or mean ± standard deviation (SD). BMI, body mass index; VAS, visual analog scale; IADL, instrumental activities of daily living. Age was calculated as 2025 minus year of birth; BMI and pain categories were recalculated from continuous values. The IADL score groups were used for descriptive display and do not represent validated severity thresholds. Percentages may not total 100% because of rounding.

**Table 2:** Univariable linear regression analysis for the Barthel Index (n = 184).

Predictor	B	95% CI Lower	95% CI Upper	R^2^	*p*
Age (years)	−0.752	−1.077	−0.427	0.127	<0.001
BMI (kg/m^2^)	−0.060	−0.817	0.697	0.000	0.875
Secondary education or higher	7.265	1.954	12.575	0.044	0.008
Urban residence	−0.582	−6.712	5.548	0.000	0.852
Hypertension	−3.719	−8.709	1.271	0.012	0.143
Diabetes mellitus	−5.124	−14.125	3.876	0.011	0.263
Corticosteroid use	−5.776	−20.456	8.904	0.006	0.439
Undocumented analgesic product use	−2.276	−7.170	2.617	0.005	0.360
Pain intensity (VAS)	−8.115	−9.277	−6.953	0.608	<0.001
T-score, femoral neck	4.273	1.475	7.071	0.043	0.003
T-score, lumbar spine	4.386	1.055	7.717	0.042	0.010
Thoracic vs. lumbar fracture	1.670	−5.141	8.482	0.002	0.629
Thoracolumbar vs. lumbar fracture	−7.487	−12.804	−2.169	0.046	0.006
Two vs. one fractured vertebra	−0.179	−6.424	6.065	0.000	0.955
≥3 vs. one fractured vertebra	−8.102	−13.451	−2.753	0.053	0.003
Menopause duration (years)	−0.573	−0.838	−0.309	0.099	<0.001

B, unstandardized regression coefficient; CI, confidence interval; R^2^, coefficient of determination; VAS, visual analog scale. Confidence intervals and *p* values use HC3 heteroskedasticity-robust standard errors. Reference categories: primary education, rural residence, no for binary clinical variables, lumbar fracture location, and one fractured vertebra. All *p* values are two-sided.

**Table 3:** Univariable linear regression analysis for the Lawton IADL scale (n = 184).

Predictor	B	95% CI Lower	95% CI Upper	R^2^	*p*
Age (years)	−0.127	−0.162	−0.093	0.237	<0.001
BMI (kg/m^2^)	0.049	−0.050	0.147	0.006	0.330
Secondary education or higher	1.189	0.548	1.830	0.076	<0.001
Urban residence	−0.027	−0.724	0.670	0.000	0.940
Hypertension	−0.401	−1.033	0.231	0.009	0.213
Diabetes mellitus	−0.311	−1.285	0.664	0.003	0.530
Corticosteroid use	−0.251	−1.953	1.452	0.001	0.772
Undocumented analgesic product use	−0.370	−0.979	0.239	0.008	0.233
Pain intensity (VAS)	−0.840	−0.979	−0.701	0.424	<0.001
T-score, femoral neck	0.674	0.326	1.023	0.070	<0.001
T-score, lumbar spine	0.651	0.268	1.034	0.061	<0.001
Thoracic vs. lumbar fracture	0.203	−0.641	1.047	0.002	0.636
Thoracolumbar vs. lumbar fracture	−0.871	−1.520	−0.222	0.040	0.009
Two vs. one fractured vertebra	0.207	−0.517	0.931	0.002	0.573
≥3 vs. one fractured vertebra	−1.082	−1.718	−0.446	0.062	<0.001
Menopause duration (years)	−0.101	−0.131	−0.071	0.201	<0.001

B, unstandardized regression coefficient; CI, confidence interval; R^2^, coefficient of determination; IADL, instrumental activities of daily living; VAS, visual analog scale. Confidence intervals and *p* values use HC3 heteroskedasticity-robust standard errors. Reference categories: primary education, rural residence, no for binary clinical variables, lumbar fracture location, and one fractured vertebra. All *p* values are two-sided.

**Table 4:** Multivariable linear regression analysis for the Barthel Index (n = 184).

Predictor	B	SE	β	95% CI	VIF	*p*
Age (years)	−0.458	0.246	−0.217	−0.944 to 0.028	4.41	0.065
BMI (kg/m^2^)	−0.675	0.299	−0.134	−1.265 to −0.084	1.35	0.025
Secondary education or higher	0.636	1.873	0.018	−3.061 to 4.334	1.19	0.734
Urban residence	2.141	2.260	0.053	−2.321 to 6.603	1.18	0.345
Hypertension	−1.310	1.920	−0.038	−5.100 to 2.481	1.28	0.496
Diabetes mellitus	−2.374	3.090	−0.048	−8.475 to 3.726	1.20	0.443
Corticosteroid use	−0.521	4.747	−0.007	−9.893 to 8.851	1.20	0.913
Undocumented analgesic product use	2.938	1.929	0.088	−0.871 to 6.747	1.31	0.130
Pain intensity (VAS)	−7.996	0.779	−0.769	−9.534 to −6.459	1.33	<0.001
T-score, femoral neck	−0.918	1.296	−0.045	−3.477 to 1.642	1.72	0.480
T-score, lumbar spine	0.281	1.365	0.013	−2.413 to 2.975	1.49	0.837
Thoracic vs. lumbar fracture	−2.104	2.648	−0.051	−7.332 to 3.124	1.27	0.428
Thoracolumbar vs. lumbar fracture	−1.354	2.609	−0.039	−6.505 to 3.798	2.58	0.605
Two vs. one fractured vertebra	−2.947	2.442	−0.073	−7.767 to 1.874	1.52	0.229
≥3 vs. one fractured vertebra	−0.708	2.897	−0.020	−6.428 to 5.012	3.03	0.807
Menopause duration (years)	0.166	0.205	0.091	−0.239 to 0.571	3.93	0.419

B, unstandardized regression coefficient; SE, HC3 heteroskedasticity-robust standard error; β, standardized regression coefficient; CI, confidence interval; VIF, variance inflation factor; VAS, visual analog scale. Reference categories: primary education, rural residence, no for binary clinical variables, lumbar fracture location, and one fractured vertebra. Model R^2^ = 0.661; adjusted R^2^ = 0.628.

**Table 5:** Multivariable linear regression analysis for the Lawton IADL scale (n = 184).

Predictor	B	SE	β	95% CI	VIF	*p*
Age (years)	−0.082	0.031	−0.314	−0.143 to −0.022	4.41	0.008
BMI (kg/m^2^)	−0.016	0.042	−0.026	−0.099 to 0.066	1.35	0.695
Secondary education or higher	0.422	0.274	0.098	−0.118 to 0.963	1.19	0.125
Urban residence	0.127	0.292	0.025	−0.449 to 0.704	1.18	0.663
Hypertension	0.003	0.261	0.001	−0.512 to 0.518	1.28	0.991
Diabetes mellitus	−0.024	0.408	−0.004	−0.830 to 0.782	1.20	0.954
Corticosteroid use	0.009	0.654	0.001	−1.281 to 1.300	1.20	0.989
Undocumented analgesic product use	0.008	0.278	0.002	−0.541 to 0.557	1.31	0.977
Pain intensity (VAS)	−0.694	0.077	−0.538	−0.845 to −0.542	1.33	<0.001
T-score, femoral neck	−0.147	0.195	−0.057	−0.532 to 0.238	1.72	0.452
T-score, lumbar spine	−0.024	0.187	−0.009	−0.392 to 0.345	1.49	0.900
Thoracic vs. lumbar fracture	−0.252	0.354	−0.049	−0.952 to 0.447	1.27	0.477
Thoracolumbar vs. lumbar fracture	−0.133	0.372	−0.031	−0.867 to 0.601	2.58	0.721
Two vs. one fractured vertebra	−0.069	0.317	−0.014	−0.695 to 0.556	1.52	0.827
≥3 vs. one fractured vertebra	−0.199	0.431	−0.046	−1.050 to 0.652	3.03	0.646
Menopause duration (years)	−0.003	0.024	−0.012	−0.049 to 0.044	3.93	0.909

B, unstandardized regression coefficient; SE, HC3 heteroskedasticity-robust standard error; β, standardized regression coefficient; CI, confidence interval; VIF, variance inflation factor; IADL, instrumental activities of daily living; VAS, visual analog scale. Reference categories: primary education, rural residence, no for binary clinical variables, lumbar fracture location, and one fractured vertebra. Model R^2^ = 0.531; adjusted R^2^ = 0.486.

**Table 6:** Ordered-logistic regression sensitivity analyses.

Predictor	BI OR	BI 95% CI	BI *p*	IADL OR	IADL 95% CI	IADL *p*
Age (years)	0.603	0.285–1.277	0.186	0.421	0.222–0.800	0.008
BMI (kg/m^2^)	0.687	0.443–1.065	0.093	0.891	0.644–1.233	0.486
Secondary education or higher	1.286	0.556–2.971	0.557	1.555	0.774–3.122	0.215
Urban residence	1.739	0.571–5.302	0.330	0.988	0.457–2.139	0.976
Hypertension	0.312	0.120–0.808	0.016	1.011	0.519–1.968	0.974
Diabetes mellitus	0.522	0.148–1.842	0.312	0.788	0.274–2.269	0.658
Corticosteroid use	1.546	0.156–15.346	0.710	1.126	0.255–4.971	0.876
Undocumented analgesic-product use	4.169	1.742–9.979	0.001	1.035	0.474–2.262	0.931
Pain intensity (VAS)	0.034	0.016–0.072	<0.001	0.186	0.120–0.288	<0.001
T-score, femoral neck	1.002	0.641–1.568	0.992	0.867	0.579–1.300	0.490
T-score, lumbar spine	1.096	0.695–1.728	0.693	0.943	0.666–1.335	0.741
Thoracic vs. lumbar fracture	0.661	0.250–1.743	0.402	0.844	0.298–2.393	0.749
Thoracolumbar vs. lumbar fracture	0.525	0.188–1.463	0.218	1.002	0.390–2.571	0.997
Two vs. one fractured vertebra	0.583	0.224–1.514	0.268	0.611	0.262–1.425	0.254
≥3 vs. one fractured vertebra	1.674	0.519–5.400	0.388	0.562	0.176–1.795	0.331
Menopause duration (years)	1.231	0.590–2.567	0.580	0.873	0.505–1.509	0.627

ORs indicate the odds of a higher functional category or score. BI was modeled using four ordered categories (≤60, 61–90, 91–99, and 100). IADL was modeled using the exact ordered scores 0–8. Age, BMI, VAS, T-scores, and menopause duration were standardized; their ORs represent a one-standard-deviation increase. Binary predictors compare the stated category with the reference category used in the primary models.

**Table 7:** Fracture coefficients in multivariable linear models with and without VAS pain intensity.

Outcome	Fracture Variable	B with VAS	*p* with VAS	B Without VAS	*p* Without VAS
Barthel Index	Thoracic vs. lumbar	−2.104	0.428	−0.957	0.814
Barthel Index	Thoracolumbar vs. lumbar	−1.354	0.605	−4.276	0.223
Barthel Index	Two vs. one fractured vertebra	−2.947	0.229	−2.715	0.513
Barthel Index	≥3 vs. one fractured vertebra	−0.708	0.807	−4.632	0.251
Lawton IADL	Thoracic vs. lumbar	−0.252	0.477	−0.153	0.711
Lawton IADL	Thoracolumbar vs. lumbar	−0.133	0.721	−0.386	0.365
Lawton IADL	Two vs. one fractured vertebra	−0.069	0.827	−0.049	0.908
Lawton IADL	≥3 vs. one fractured vertebra	−0.199	0.646	−0.539	0.262

All models contained the same prespecified covariates, except that the comparison model omitted VAS. HC3 robust *p* values are shown. Lumbar fracture location and one fractured vertebra were the reference categories.

**Table 8:** Exploratory nonlinearity, interaction, and influence analyses.

Analysis	Barthel Index	Lawton IADL	Interpretation
Age squared, HC3 *p*	0.010	<0.001	Evidence of nonlinearity
BMI squared, HC3 *p*	0.220	0.060	No BI nonlinearity; borderline IADL evidence
VAS squared, HC3 *p*	<0.001	<0.001	Evidence of nonlinearity
Joint VAS × fracture interactions, *p*	0.360	0.587	No evidence of effect modification
Observations with Cook’s distance > 4/n	12	10	Sensitivity analysis performed
Maximum Cook’s distance	0.146	0.065	No observation approached 1
VAS after excluding influential observations	B = −7.004; *p* <0.001	B = −0.724; *p* <0.001	Strong association preserved
BMI/age after excluding influential observations	BMI B = −0.875; *p* < 0.001	Age B = −0.081; *p* = 0.007	Primary domain-specific findings preserved

Each quadratic term was added separately to the full prespecified linear model after centering its linear component. The interaction test jointly assessed VAS interactions with thoracic location, thoracolumbar location, two fractures, and three or more fractures. Influence sensitivity analyses excluded observations exceeding 4/n.

## Data Availability

The datasets generated and/or analyzed during the current study are not publicly available because they contain clinical data from human participants but are available from the corresponding author upon reasonable request and subject to institutional and ethical approval.

## Abbreviations

The following abbreviations are used in this manuscript:

ADL	Activities of daily living
BI	Barthel Index
BMI	Body mass index
BMD	Bone mineral density
CI	Confidence interval
DXA	Dual-energy X-ray absorptiometry
HC3	Heteroskedasticity-consistent covariance estimator, type 3
IADL	Instrumental activities of daily living
OR	Odds ratio
OVF	Osteoporotic vertebral fracture
SD	Standard deviation
VAS	Visual analog scale
VIF	Variance inflation factor
